# Transarterial chemoembolization plus or minus intravenous bevacizumab in the treatment of hepatocellular cancer: A pilot study

**DOI:** 10.1186/1471-2407-12-16

**Published:** 2012-01-14

**Authors:** Carolyn D Britten, Antoinette S Gomes, Zev A Wainberg, David Elashoff, Rafael Amado, Yan Xin, Ronald W Busuttil, Dennis J Slamon, Richard S Finn

**Affiliations:** 1 Division of Hematology/Oncology, Department of Medicine, David Geffen School of Medicine at the University of California, Los Angeles, 2825 Santa Monica Blvd., Suite 200, Santa Monica, CA 90404-2429, USA; 2 Departments of Medicine and Radiologic Science, David Geffen School of Medicine at the University of California, Los Angeles, Box 957437, Ronald Reagan Medical Center, Suite 2125, Los Angeles, CA 90095-7437, USA; 3 Department of Medicine, David Geffen School of Medicine at the University of California, Los Angeles, 10940 Wilshire Blvd., Suite 1223, Los Angeles, CA 90095-7362, USA; 4 Genentech, Inc., 1 DNA Way, MS 46-3A, South San Francisco, CA, USA; 5 Department of Surgery, Chairman's Office, David Geffen School of Medicine at the University of California, Los Angeles, Box 957430, 757 Westwood Plaza, Suite 8236, Los Angeles, CA 90095-7430, USA

## Abstract

**Background:**

Stimulation of vascular endothelial growth factor (VEGF) has been observed following transarterial chemoembolization (TACE) in hepatocellular cancer (HCC) and may contribute to tumor regrowth. This pilot study examined whether intravenous (IV) bevacizumab, a monoclonal antibody against VEGF, could inhibit neovessel formation after TACE.

**Methods:**

30 subjects with HCC undergoing TACE at a single academic institution were randomized with a computer-generated allocation in a one to one ratio to either bevacizumab at a dose of 10 mg/kg IV every 14 days beginning 1 week prior to TACE (TACE-BEV arm) or observation (TACE-O arm). Angiography was performed with TACE at day 8, and again at weeks 10 and 14. Repeat TACE was performed at week 14 if indicated. TACE-BEV subjects were allowed to continue bevacizumab beyond week 16. TACE-O subjects were allowed to cross-over to bevacizumab at week 16 in the setting of progressive disease. The main outcome measure was a comparison of neovessel formation by serial angiography. Secondary outcome measures were progression free survival (PFS) at 16 weeks, overall survival (OS), bevacizumab safety, and an analysis of VEGF levels before and after TACE with and without bevacizumab.

**Results:**

Among the 30 subjects enrolled, 9 of 15 randomized to the TACE-O arm and 14 of 15 randomized to the TACE-BEV arm completed all 3 angiograms. At week 14, 3 of 9 (33%) TACE-O subjects and 2 of 14 (14%) TACE-BEV subjects demonstrated neovascularity. The PFS at 16 weeks was 0.19 in the TACE-O arm and 0.79 in the TACE-BEV arm (*p *= 0.021). The median OS was 61 months in the TACE-O arm and 49 months in the TACE-BEV arm (*p *= 0.21). No life-threatening bevacizumab-related toxicities were observed. There were no substantial differences in bevacizumab pharmacokinetics compared to historical controls. Bevacizumab attenuated the increase in VEGF observed post-TACE.

**Conclusions:**

IV bevacizumab was well tolerated in selected HCC subjects undergoing TACE, and appeared to diminish neovessel formation at week 14.

**Trial registration:**

ClinicalTrials.gov NCT00049322.

## Background

Hepatocellular cancer (HCC) is a global health concern, with an incidence exceeding 500,000 new cases per year worldwide [1]. In the United States, HCC is one of the few cancers with rising rates of incidence and mortality [2]. This increasing incidence has been directly linked to hepatitis C, and indirectly linked, through studies in obesity and diabetes, to non-alcoholic steatohepatitis (NASH) [3-5].

The management of hepatocellular cancer (HCC) is dictated by the degree of underlying liver dysfunction, the burden of malignancy, and the patient's performance status [6]. Within this framework, patients are stratified into treatment groups, including resection, percutaneous ablation, transarterial chemoembolization (TACE), orthotopic liver transplantation (OLT), systemic therapy, and/or supportive care. TACE is generally employed in the treatment of large (> 3 cm) or multifocal HCC confined to the liver, in the context of preserved liver function [6]. Although TACE is a palliative procedure by itself, TACE may be used to control disease in patients awaiting curative OLT.

TACE takes therapeutic advantage of the liver's dual blood supply: hepatocellular cancer cells are preferentially supplied by branches of the hepatic artery, whereas hepatocytes are preferentially supplied by branches of the portal vein [6]. By infusing chemotherapeutic agents directly into vessels supplying the tumor, and subsequently obstructing these vessels with an embolization material, the HCC receives prolonged exposure to the chemotherapeutic agent, and is deprived of its' blood supply. This technique has demonstrated its ability to improve overall survival compared to supportive care in a meta-analysis [7], and in randomized phase III clinical trials [8,9]. Ultimately, however, TACE fails due to incomplete embolization, partial recanalization, and/or induction of neovascularization [10].

Vascular endothelial growth factor (VEGF) is a potent regulator of neovascularization that appears to play a role in HCC [11]. Serum VEGF levels are higher in patients with HCC than in patients with benign liver lesions or healthy controls [12,13]. Also, higher serum or plasma VEGF levels predict clinicopathologic features of HCC, including increased tumor size, presence of distant metastases and/or vascular invasion, and advanced stage [12-14]. Patients with higher pre-operative serum VEGF levels have decreased disease-free and overall survival [13,15], and patients with higher pre-procedure VEGF levels are less likely to respond to TACE [12,16]. Finally, TACE appears to upregulate VEGF through an induction of tumor anoxia and ischemia [10,12,17]. This evidence supports the investigation of anti-VEGF therapy in the treatment of HCC.

Given the role of VEGF in HCC, and the changes in VEGF associated with TACE, this pilot study was designed to test the hypothesis that therapy directed against VEGF may improve upon the results observed with TACE alone. Bevacizumab, a humanized monoclonal antibody against VEGF was chosen over one of the multi-targeted anti-angiogenic agents because bevacizumab could more directly test the VEGF hypothesis. Also, in an orthotopic HCC Hep3B model, bevacizumab significantly decreased tumor microvessel density, decreased human serum α-fetoprotein (AFP), and prolonged the time to progression in treatment mice compared to control mice [18]. In this investigator-initiated pilot study, patients who were scheduled to undergo TACE were randomized to either observation (TACE-O) or intravenous bevacizumab (TACE-BEV). All patients underwent sequential angiograms, providing a unique opportunity to study neovascularity in real time. The principal objective was to compare neovessel formation at 10 and 14 weeks in patients treated with TACE plus or minus intravenous bevacizumab. The secondary objectives were to: i) assess progression free survival (PFS) at 16 weeks; ii) determine overall survival (OS); iii) describe the toxicities of bevacizumab in liver disease; iv) evaluate the pharmacokinetics (PK) of bevacizumab in liver disease; and v) measure VEGF before and after TACE with and without bevacizumab.

## Methods

### Patient selection

Eligible subjects at a single academic institution had HCC by virtue of either: i) the European Association for the Study of the Liver (EASL) diagnostic imaging criteria [19]; or ii) biopsy. All subjects were clinical candidates for TACE with at least one lesion ≥ 3 cm and no lesion ≥ 15 cm, and no more than three lesions total. Tumor location had to permit embolization of all tumor nodules on initial TACE. Subjects awaiting OLT were eligible if their model for end-stage liver disease (MELD) priority score was < 28 points at entry. The MELD score cutoff was based on a median MELD score of 30 at the time of transplant at our institution during the period that the study was conducted. This allowed subjects to complete the three scheduled angiograms before being withdrawn from study.

Subjects were excluded if they had Child's class C liver dysfunction, Eastern Cooperative Oncology Group (ECOG) Performance Status (PS) >2, bilirubin >2.5 mg/dL, INR > 1.5, extrahepatic disease, or thrombosis of the main portal vein. Subjects were excluded if they demonstrated contraindications to bevacizumab. Initially, subjects were excluded if they had platelets < 100,000/μL. After the first three subjects enrolled in the TACE-BEV arm completed week 10 without significant bleeding (defined as bleeding requiring transfusion), additional subjects were excluded if they had platelets < 75,000/μL. After safety was confirmed using the 75,000/μL platelet cut-off, the protocol was amended in consultation with the Food and Drug Administration (FDA) to exclude subjects with platelets < 60,000/μL, in an effort to improve accrual. The protocol was approved by the Medical Institutional Review Board at the University of California, Los Angeles. Written informed consent was obtained and the study was conducted according to federal and institutional guidelines, observing the standards set by the Helsinki Declaration.

### Study design and treatment procedures

**Figure 1:**
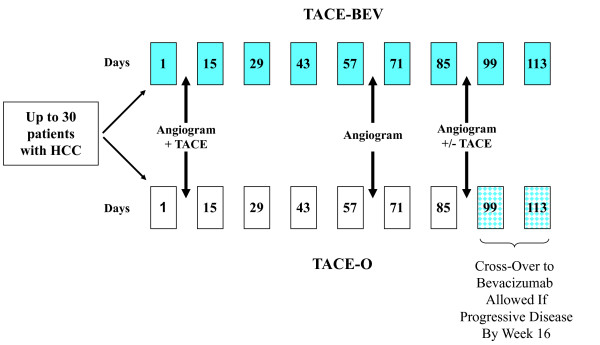
**Study design**.

The study design is depicted in Figure 1. A one to one randomization was performed with a computer-generated allocation, distributing 30 subjects to either TACE-O or TACE-BEV.

Hepatic angiograms were scheduled at day 8, week 10, and week 14. The day 8 procedure included TACE of all lesions. The week 10 and 14 angiograms were performed to assess tumor vascularity. Two observation points were chosen because it was unknown how long it would take to develop a treatment effect, and 10 and 14 weeks were specifically selected to approximate the 2 to 3 months separating the first and second scheduled TACE procedures in previous clinical trials (8,9). TACE was not performed during the week 10 angiogram, justified by the opportunity for TACE at week 14. At week 14, a second TACE was performed if: i) a previously embolized feeding vessel had recanalized; or ii) there was residual tumor blush in a treated area, and a feeding vessel was visualized; or iii) new lesions had developed that were amenable to TACE.

TACE was performed using doxorubicin 25 mg/m^2 ^(emulsified with lipiodol, 10-12 mL), cisplatin 50 mg/m^2^, and mitomycin-C 5 mg/m^2^, with Embosphere^® ^microspheres (Biosphere Medical, Rockland, MA). Chemotherapy and the embolic agent were administered through the hepatic artery to segmental and/or subsegmental branches feeding the tumor. Stump occlusion of segmental or subsegmental feeding branches was performed with microfibrillar collagen (Avitene, Davol, Inc., Cranston RI) as needed to achieve stasis. Following TACE, subjects were admitted to the hospital for at least 24 h, where they received hydration, pain control, and antiemetics as needed. Subjects also received metronidazole and ciprofloxacin for 7 days after TACE.

Subjects randomized to TACE-BEV received bevacizumab 10 mg/kg intravenously (IV) on day 1, one week prior to the first TACE. Post-TACE, bevacizumab administration resumed at 10 mg/kg every 2 weeks, as long as serum transaminases had returned to pre-TACE levels, or within normal range. Subjects receiving bevacizumab with no signs of progressive disease were allowed to continue bevacizumab therapy until: i) they experienced unacceptable toxicity; or ii) they developed tumor progression (as defined below); or iii) their MELD score increased to > 28 points; or iv) they requested discontinuation of drug.

Subjects randomized to TACE-O were eligible to cross-over to bevacizumab at week 16 if they had evidence of progressive disease. Subjects who crossed over were required to meet the original eligibility criteria, and the criteria for bevacizumab administration status post TACE, as outlined above.

### Pretreatment and follow-up studies

All subjects underwent baseline procedures including a history and physical, 12-lead electrocardiogram, and tumor assessment with triple phase computed tomography scan (CT) and/or contrast enhanced magnetic resonance imaging scan (MRI) of the liver within 4 weeks of starting protocol treatment. Additional staging investigations were performed at the discretion of the investigator. Subjects with documented grade III varices, or a history of upper gastrointestinal bleeding, were required to undergo endoscopic evaluation prior to study treatment. Within 2 weeks of starting protocol treatment, a physical exam, complete blood count (CBC), comprehensive metabolic panel (CMP), international normalized ratio (INR), urinalysis, and AFP were performed.

The first 16 weeks constituted the core treatment period. CBCs and CMPs were obtained daily for 3 days post-TACE, 1 week post-TACE, 2 weeks post-TACE, and every 4 weeks thereafter. This CBC and CMP schedule was repeated after a second TACE. An INR was obtained on day 3 post-TACE, then 3 weeks post-TACE, and every 4 weeks thereafter. This INR schedule was repeated after a second TACE. AFP was obtained every 2 weeks for the first 16 weeks, then every 4 weeks thereafter. Urinalysis or urine dipstick to screen for proteinuria was performed every 2 weeks. Physical exams were performed within 2 weeks prior to all angiograms and within 2 weeks following all angiograms. Toxicities were graded according to the National Cancer Institute common toxicity criteria (CTC), version 3.0. Follow-up CT or MRI was performed at weeks 8 and 16.

Subjects who received bevacizumab after week 16 were evaluated at least monthly with a physical exam, CBC, CMP, INR, and AFP. Urinalysis or urine dipstick was performed every 2 weeks. These safety evaluations were also performed at 30 and 60 days after the last dose of bevacizumab. CT or MRI for disease assessment was performed every 8 weeks while on bevacizumab.

Subjects who completed the 16 week core phase plus or minus the bevacizumab continuation phase were followed per institutional practice. All subjects were assessed for survival every 3 months.

### Efficacy

Angiograms were assessed for changes in vascularity using the following parameters: 1) neovessel formation; 2) recanalization; 3) development of collaterals; and 4) number of vessels. All changes in vascularity were assessed with the catheter in the common hepatic artery, to allow standardized visualization. Neovessels, defined as fine vessels with disordered arborization patterns, were scored at weeks 10 and week 14 as follows: 0, no evidence of neovessel formation; 1+, some evidence of neovessel formation; 2+, obvious vessel formation (small vessels); and 3+, obvious vessel formation (small and large vessels). Recanalization was defined as the restoration of flow in a previously occluded vessel. Collateral vessels were characterized as small well formed vessels communicating between non-embolized and embolized vessels. The number of vessels was determined retrospectively in an unblinded fashion at the end of the study for all subjects who completed 2 or more angiograms by the trial interventional radiologist. This angiographic vessel count was performed using a single field measuring 2.43 cm in diameter (the size of an American quarter) at the site with the highest tumor vessel density. Although the arteriograms were obtained in several projections, all images were graded in anteroposterior projection. Accurate comparisons between studies and reproduction of magnification factors were achieved by performing the series of arteriograms in each individual subject with the same field size, object film distance, external fiducial marker, and contrast injection parameters as on their first TACE arteriogram. Vessel counts at 10 and 14 weeks were expressed as percent change from baseline. Vessel counts were compared between groups using the Wilcoxon rank sum test.

Cross-sectional imaging was considered along with angiography when assessing disease progression. Progressive disease was defined as any one of the following: an increase in the sum of the bidimensional products of all known disease by at least 25% by cross-sectional imaging, an increase in enhancement of a previously treated lesion by cross-sectional imaging, the appearance of a new lesion, or evidence of neovascularization by angiography at week 14. The angiographic demonstration of recanalization and/or the development of collaterals without neovascularization were not considered progressive disease. An objective response rate by cross-sectional imaging was not determined because most subjects on this investigator-initiated study were evaluated by CT, and lipiodol obscured tumor measurements by CT.

Progression free survival (PFS) at 16 weeks (end of the core phase) and overall survival (OS) were estimated by the Kaplan-Meier method, using JMP 6 statistical software (SAS institute, Cary, NC). The PFS times were censored based on the minimum time of four possible events: 1) last disease assessment; 2) orthotopic liver transplant; 3) second TACE if performed in the absence of progressive disease (e.g. embolization of a recanalized vessel without neovessel formation); 4) administration of a test dose of chemoembolization material, per institutional practice. OS was calculated based on intention to treat, and cross-over subjects were kept in the observation arm for the purpose of calculating OS. PFS and OS were compared between groups using log-rank tests.

### Bevacizumab pharmacokinetics

Trough and peak blood samples were drawn from subjects randomized to the TACE-BEV arm for infusions one through five, and seven. Additional samples were drawn immediately prior to chemoembolization, and 72 h after chemoembolization. Bevacizumab serum concentrations were determined by an enzyme-linked immunosorbent assay (ELISA) with a minimum quantifiable concentration (MQC) in neat serum of 0.078 μg/mL [20]. Bevacizumab concentration data was characterized by descriptive statistics at each time-point, with data described as means and 95% confidence intervals. To assess the difference of bevacizumab PK between the HCC subjects on this study and other oncology subjects, predicted concentrations for 500 patients were simulated using a population PK model of bevacizumab established on data from eight Phase I/II/III clinical trials (on file at Genentech). In this population PK model, significant PK covariates include weight, gender, and serum albumin on clearance (CL) and volume of distribution in the central compartment (V1); total protein on clearance; and baseline tumor burden on V1. Mean concentrations and associated 95% confidence intervals were calculated from these simulations and compared to the study subjects. These simulations were performed in NONMEM version VI beta, level 1.0 (LLC, Globomax, Ellicott City, MD).

### Serum VEGF levels

Blood samples were obtained at the following time points: immediately prior to TACE; 1, 24, 48, and 72 hours after TACE; and 7, 15, and 21 days after TACE. VEGF-A was measured using an ELISA kit specific for human VEGF (R&D Systems, Minneapolis, MN, USA). Data was analyzed as the average for each timepoint and presented as fold-change from baseline. This was done for all subjects in the observation and bevacizumab arms that had at least one sample available for analysis. A one-sided *t*-test was performed for each timepoint.

## Results

### Study population

**Figure 2:**
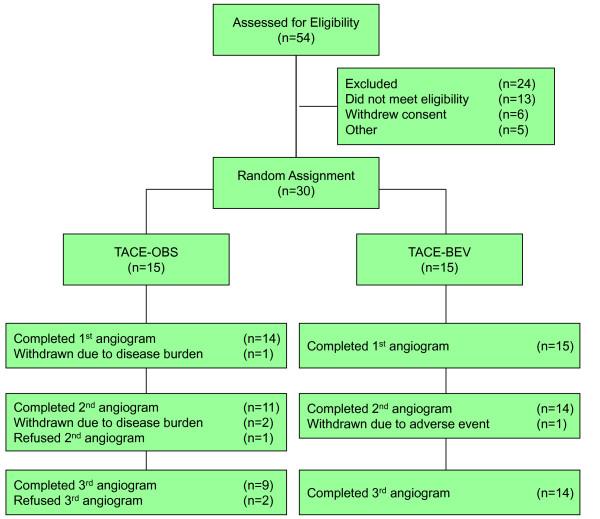
**Disposition of subjects**.

Between August 2003 and October 2008, 54 subjects were screened and 30 subjects were randomly assigned, as illustrated in Figure 2. Subject characteristics are outlined in Table 1.

**Table 1:** Subject Characteristics

Characteristic	Number of Subjects	
	
	TACE-O	TACE-BEV
Total	15	15

Median Age (Range)	[58 (49-75)]	[61 (50-79)]

Gender		

Male	12	13

Female	3	2

ECOG Performance Status		

0	13	11

1	2	4

Child-Pugh Class		

A	15	13

B	0	2

Underlying Liver Disease^a,b^		

Hepatitis B	2	4

Hepatitis C	9	7

Ethanol-Related	4	1

Cryptogenic	3	3

Hemachromatosis	0	1

Median (Range) Baseline Labs		

AFP, ng/mL	[14.9 (1.8-42,550)]	[360 (1.7-9,097)]

Total Bilirubin, mg/dL	[0.9 (0.6-1.4)]	[1.1 (0.5-1.7)]

Albumin, mg/dL	[4 (2.9-4.4)]	[3.7 (2.8-4.4)]

Tumor Characteristics		

Mean Tumor Burden^c^, cm (± SD)	[7.4 (± 2.9)]	[6.5 (± 2.0)]

Multifocal Tumor	4	4

Vascular Invasion	1	0

BCLC Stage		

A	3	1

B	10	10

C^d^	2	4

Previous Anti-Cancer Therapy		

Resection Alone	1	0

Resection and TACE	0	1

None	14	14

^a ^On the TACE-O arm, 2 subjects had hepatitis C and ethanol-related disease, and 1 subject had hepatitis B and C co-infection.

^b ^On the TACE-BEV arm, 1 subject had hepatitis C and hemachromatosis.

^c ^Tumor burden defined as the sum of the longest unidimensional measurements.

^d ^All subjects with BCLC stage C disease were characterized as stage C due to an ECOG PS of 1.

### Angiogram findings

As demonstrated in Figure 2, three TACE-O subjects withdrew prior to completing study procedures due to the volume of disease: one before and two after the first TACE. An additional three TACE-O subjects withdrew at their own request before completing all three angiograms. In contrast, only one TACE-BEV subject was withdrawn prior to study completion, due to liver dysfunction experienced after day 8 TACE.

**Figure 3:**
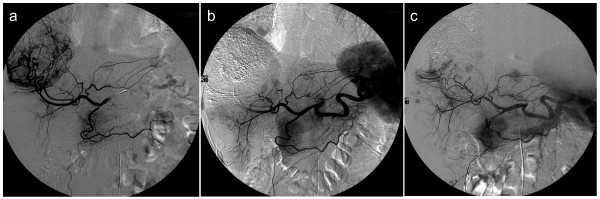
**Serial angiograms from a subject on the observation arm demonstrating neovascularity: **a**) pre-TACE angiogram on Day 8 demonstrating tumor blush; **b**) post-TACE angiogram on Day 8 demonstrating successful embolization of vessels supplying the tumor; and **c**) Week 10 angiogram demonstrating 2+ neovascularity and recanalization**.

Regarding the primary endpoint of neovessel formation at weeks 10 and 14, all angiograms were reviewed in real time, with decision for further treatment based on real time assessments. At week 10, 4 of 11 (36%) TACE-O and 4 of 14 (29%) TACE-BEV subjects demonstrated neovascularity. At week 14, 3 of 9 (33%) TACE-O and 2 of 14 (14%) TACE-BEV subjects demonstrated neovascularity. Of note, the TACE-BEV subject with 3+ neovascularity at week 14 had neovessels arising from a persistent vessel supplying the tumor that had not been treated during the first TACE. Figure 3 depicts serial angiograms demonstrating neovascularity from a subject on the observation arm.

Other angiographic findings included collateralization and recanalization. Collateralization was present in 3 of 11 (27%) TACE-O subjects and 3 of 14 (21%) TACE-BEV subjects at week 10, and in 1 of 9 (11%) TACE-O subjects and 2 of 14 (14%) TACE-BEV subjects at week 14. Recanalization was present in 3 of 11 (27%) TACE-O subjects and 3 of 14 (21%) TACE-BEV subjects at week 10, and in 3 of 9 (33%) TACE-O subjects and 4 of 14 (29%) of TACE-BEV subjects at week 14. All subjects with recanalization and/or collateralization at week 14 underwent TACE with the exception of one TACE-BEV subject, who had recanalization without active tumor confirmed by a lack of ethiodol uptake.

In total, 4 of 9 (44%) TACE-O subjects and 6 of 15 (40%) TACE-BEV subjects underwent TACE at week 14. Among the 6 TACE-BEV subjects undergoing TACE at week 14 were two subjects with persistent vessels missed during the first TACE, one subject with recanalization, one subject with recanalization and collateralization, one subject with collateralization and neovessel formation, and one subject without evidence of active tumor who received a test dose of chemoembolization material in the vessel stump.

In an attempt to quantify the angiographic findings, a vessel count was performed in all subjects undergoing more than one angiogram, with week 10 and week 14 results compared to baseline. At week 10, the mean (± standard deviation) percent change from baseline was -63.4 ± 57.7% for the TACE-O arm, and -60.4 ± 31.2% for the TACE-BEV arm, whereas at week 14, the values were -56.5 ± 54.6% and -61.1 ± 23.3%, respectively. There was no significant difference between the treatment groups by this measure.

### Survival and follow-up

**Figure 4:**
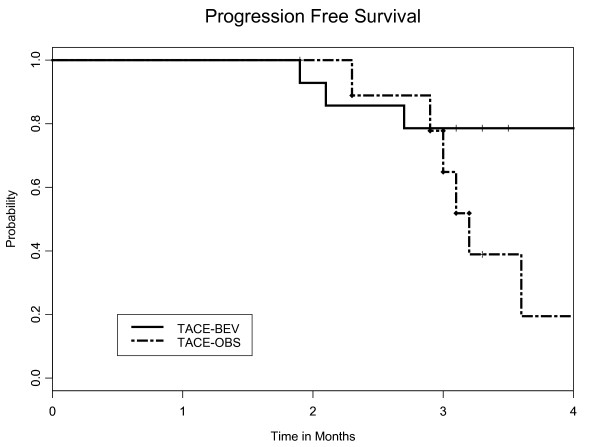
**Kaplan-Meier plot for progression free survival (PFS)**. The PFS at 16 weeks was 0.79 in the TACE-BEV arm (solid line) and 0.19 in the TACE-O arm (dashed line), with a *p*-value of 0.021

**Figure 5:**
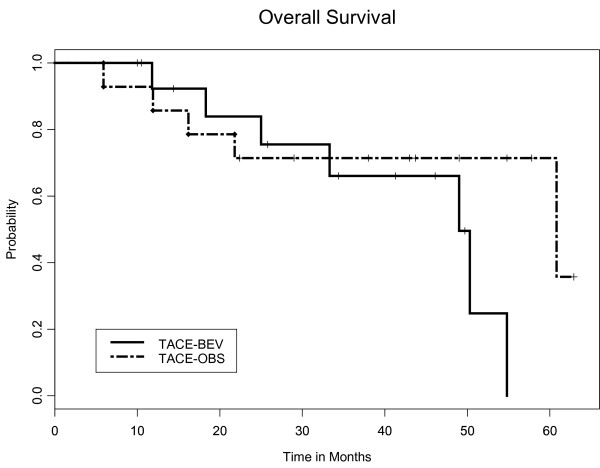
**Kaplan-Meier plot for overall survival (OS)**. The median OS was 49 months in the TACE-BEV arm (solid line) and 61 months in the TACE-O arm (dashed line), with a p-value of 0.21

The PFS at 16 weeks was 0.19 in the TACE-O arm and 0.79 in the TACE-BEV arm (*p *= 0.021), as depicted in Figure 4. The median OS was 61 months in the TACE-O arm and 49 months in the TACE-BEV arm (*p *= 0.21), depicted in Figure 5. The OS data is based on an intent-to-treat analysis, independent of cross-over status at week 16. Among the 12 subjects known to have died, ten succumbed to hepatocellular cancer and two (one in each arm) succumbed to liver failure.

Once withdrawn from study, subjects received treatment per institutional practice, including local modality therapy for recurrent disease (TACE and/or radiofrequency ablation), OLT, and chemotherapy or sorafenib (beginning in 2007) for advanced disease. 12 subjects (4 observation and 8 bevacizumab) were transplanted, with a median time from first study TACE to OLT of 11.3 (range, 3.5 to 18.1) months. Among the transplanted subjects, 10 (8 bevacizumab and 2 cross-over) received bevacizumab during the study, with a median time from the last dose of bevacizumab to OLT of 8.0 (range, 0.3 to 17.4) months.

### Safety

The most common treatment-related toxicities experienced during the first 16 weeks by the 29 subjects who received study treatment are listed by treatment arm and maximum CTC grade in Table 2. As expected, all subjects experienced elevated transaminases, and many experienced pain, pyrexia, nausea/vomiting, and fatigue. One TACE-BEV subject developed grade 4 hyperbilirubinemia after day 8 TACE, and was withdrawn from study for liver dysfunction, having received only one dose of bevacizumab. This subject was subsequently found to have spontaneous bacterial peritonitis, possibly related to a paracentesis performed just prior to study enrollment. With treatment, the liver dysfunction improved, and he was successfully re-treated with TACE off study several months later. There were no other events of severe liver dysfunction. The only clinically significant hemorrhage during the first 16 weeks occurred in a TACE-O subject who developed a grade 3 variceal bleed during week 9. Following banding and transfusion, she was able to complete the week 10 angiogram without incident.

**Table 2:** Most Common Treatment-Related Toxicities Experienced During First 16 Weeks

Toxicity	Number of Subjects by Maximum CTC Grade				Total
	
	TACE-BEV (N = 15)		TACE-O (N = 14)		
	
	Grade 1-2	Grade 3-4	Grade 1-2	Grade 3-4	
	
Anemia	3	0	5	1	9
Anorexia	5	0	4	0	9

Bleeding	8	0	0	1	9

Constipation	4	0	3	0	7

Electrolyte Abnormalities	5	4	8	1	18

Elevated Alkaline Phosphatase	4	0	3	0	7

Elevated Transaminases	1	14	3	11	29

Fatigue	9	0	8	0	17

Hyperbilirubinemia	6	2	7	0	15

Hypertension	3	3	1	3	10

Hypoalbuminemia	5	0	7	0	12

Nausea and/or Vomiting	6	0	9	0	15

Pain	9	2	12	1	24

Proteinuria	7	1	1	0	9

Pyrexia	8	0	7	0	15

Thrombocytopenia	4	3	10	0	17

Table 2 lists three events of grade 3 hypertension in each of the two treatment arms. In general, TACE-BEV subjects had sustained hypertension requiring ongoing treatment, whereas TACE-O subjects had transient hypertension during and/or immediately following TACE.

Taking the core (weeks 1 to 16) and continuation (beyond 16 weeks) phases together, a total of 19 subjects (15 TACE-BEV and 4 cross-over) received bevacizumab for a median of 4.7 months (range, 0.03 to 11.6 months). Bevacizumab-related toxicities included controllable grade 3 hypertension in 4 subjects, grade 2 to 3 proteinuria in 7 subjects, and grade 1 epistaxis in 9 subjects. Three TACE-BEV subjects experienced gastrointestinal bleeding during the continuation phase: one subject with a prior history of varices experienced grade 3 variceal bleeding within 1 week after withdrawing from study; another subject experienced grade 2 bleeding from portal hypertensive gastropathy after nearly 11 months of bevacizumab and was subsequently withdrawn from study; and a third subject experienced grade 3 bleeding secondary to gastric ulcers within 1 month after withdrawing from study. Reasons for discontinuing bevacizumab included bevacizumab-related toxicities (5 subjects), progressive disease (3 subjects), subject withdrawal (3 subjects), OLT evaluation and/or treatment (3 subjects), progressive thrombocytopenia (2 subjects), acute liver dysfunction after TACE (1 subject), or a combination of factors listed above (2 subjects).

### Pharmacokinetics

The descriptive statistics for the observed bevacizumab concentrations and the corresponding predicted concentrations from the population simulations are summarized in Table 3 for each time-point. Mean observed bevacizumab concentrations were generally less than those predicted from simulations with overlapping 95%-confidence intervals at each of the assessed time points. In addition, the clearance of bevacizumab in this study (mean 12.6 mL/h, ranging from 7.98 to 18.3 mL/h) was comparable to the clearance in general oncology patients (mean 9.75 mL/h, ranging from 3.05 to 23.1 mL/h). These data suggest that there are no substantial differences in bevacizumab PK between HCC subjects undergoing TACE and other oncology patients. However, this observation is limited by the small number of subjects enrolled on the bevacizumab arm (n = 15).

**Table 3:** Comparison of observed bevacizumab serum concentrations (unit: μg/mL) in the current study to predicted data

			Mean Concentration (95% Confidence Interval)				
		
Time		Day 1	Day 15	Day 29	Day 43	Day 57	Day 85
Peak	Study Subjects	203 (181-225)	237 (211-263)	297 (268-325)	280 (232-328)	272 (231-313)	333 (300-366)
	
	Predicted from PopPK	256 (147-405)	317 (172-497)	356 (196-569)	378 (212-616)	392 (212-628)	404 (207-664)

Trough	Study Subjects	N/A^a^	35.4 (31.5-39.4)	74.3 (66.7-82.0)	84.7 (60.4-109)	97.7 (71.3-124)	119 (95.4-142)
	
	Predicted from PopPK	N/A	58.2 (23.6-103)	95.6 (43.1-171)	122 (50.9-232)	139 (51.3-270)	156 (60.0-323)

Time		Day 8	Day 11				

Other	Study Subjects	83.2 (71.7-94.6)	60.9 (53.2-68.6)				
					
	Predicted from PopPK	78.8 (34.4-138)	67.8 (27.9-123)				

^a ^N/A is 'not applicable'.

### Serum VEGF levels

**Figure 6:**
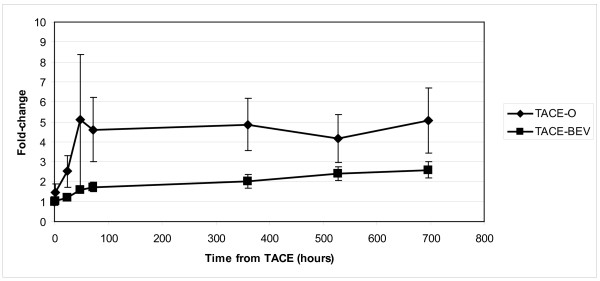
**Effect of TACE on serum VEGF levels**. Fold-change in VEGF levels over time are depicted for observation and bevacizumab arms. One-sided *t*-test values for each timepoint are as follows: 1 h, 0.053; 24 h, 0.167; 48 h, 0.161; 72 h, 0.048; 360 h, 0.039; 528 h, 0.063; 696 h, 0.06

The effects of TACE on serum VEGF levels in the presence and absence of VEGF blockade with bevacizumab are shown in Figure 6. The results are depicted as fold-change from baseline for the TACE-O and TACE-BEV arms. VEGF levels increased after TACE in both arms, however, the fold-change was higher in the TACE-O arm.

## Discussion and conclusion

This is the first clinical trial of a systemic anti-angiogenic agent used in combination with TACE. At the time that this investigator-initiated pilot trial was initiated in 2003, systemic anti-angiogenic agents were unproven in HCC. Subsequent to the initiation of this study, a molecularly-targeted approach for the treatment of advanced HCC was validated by the SHARP trial, which demonstrated an improvement in overall survival with sorafenib compared to placebo [21]. However, the use of molecularly-targeted agents in advanced disease may not take full advantage of the potential activity of this class of agents. Anti-angiogenic agents are now being tested in large multi-institutional phase III trials for subjects with early stage HCC, as an adjunct to TACE, RFA, and resection. The results of this pilot study of TACE plus or minus bevacizumab demonstrate that anti-angiogenic agents are a tolerable adjunct to TACE, and set the stage for larger clinical trials examining this approach.

TACE in HCC provides a unique opportunity to study neovascularity in real time, because angiograms are performed as part of the therapeutic procedure. In this study, angiograms were used qualitatively to determine neovascularity on a 0 to 3+ scale at 10 and 14 weeks after TACE, and with this technique, there was a trend towards decreased neovessel formation in subjects who received bevacizumab compared to those who did not. In contrast, bevacizumab did not protect against recanalization, perhaps because recanalization represents a mechanical failure of the embolization material rather than a response to tumor hypoxia. In addition to the qualitative analysis of neovessel formation, tumor blood supply was quantified by measuring blood vessel density in a representative field on the angiogram, much like microvessel density is assessed by immunohistochemisty in tumor biopsies. There was no significant difference in the angiographic blood vessel density between the two treatment arms. The failure to demonstrate a decrease in blood vessel density in the combination arm may be explained by either a true lack of benefit or by a limitation of the methodology employed. Other studies of anti-angiogenic agents in advanced HCC have examined vessel permeability, and tumor blood volume and blood flow using dynamic contrast enhanced MRI (DCE-MRI) or perfusion CT [22,23]. In recent years, there has been an increasing interest in the use of these techniques as surrogates of microvessel density as measured by immunohistochemistry [24], and there is an ongoing effort to incorporate *in vivo *assessments of tumor angiogenesis into clinical trials of anti-angiogenic agents.

The statistically significant improvement in PFS observed in the TACE-BEV arm is interpreted with caution, due to the small sample size. Progression was taken as any new sign of tumor activity, including neovascularity on angiogram, new enhancement of an existing lesion by CT or MRI, or development of a new lesion, according to institutional practice. There was a low threshold for determining progressive disease, because TACE was often used as a bridge to transplant, and earlier treatment could potentially prevent subjects from becoming ineligible for transplant due to tumor volume. The modified Response Evaluation Criteria in Solid Tumors (mRECIST) criteria for hepatocellular cancer, in which disease assessments are based on the sum of the diameters of viable disease rather than on the sum of the total diameters of the target lesions, were published after this study had begun [25]. The mRECIST criteria were not applied retrospectively because the majority of subjects on this trial were imaged with CT rather than MRI, and ethiodol used during TACE obscured the CT findings of enhancement.

There was no statistically significant difference in overall survival between the two treatment arms, with a median survival of 49 months for the TACE-BEV arm, and 61 months for the TACE-O arm. Beyond 36 months, the ability to estimate the survival curves with any precision is impaired due to small sample size and wide confidence intervals. Analysis of overall survival is further hampered by the cross-over design. Any perceived difference in overall survival cannot be attributed to bevacizumab toxicity, because there were no bevacizumab-related deaths, and all subjects succumbed to underlying disease. As a whole, the striking overall survival rates in this pilot study are undoubtedly due to the fact that 12 of the 30 subjects enrolled were ultimately transplanted.

At the initiation of this trial, there was no prior experience with bevacizumab in HCC, and there were concerns regarding the safety of bevacizumab in subjects with an increased risk of bleeding. Several layers of protection were built into the study to address this issue, including a minimum platelet count of 60 to 100,000/μL (depending on the time of enrolment), mandatory endoscopy for any subject with a prior history of grade III varices or gastrointestinal bleeding, and careful follow-up. During the core phase, none of the 15 TACE-BEV subjects developed gastrointestinal bleeding, while one TACE-O subject experienced grade 3 variceal bleeding. During the continuation phase, 3 of the 13 (9 TACE-BEV and 4 cross-over) subjects who received bevacizumab developed grade 2/3 treatment-related gastrointestinal bleeding, but no episode was life-threatening. In addition, while every effort was made to prevent subjects from receiving bevacizumab at the time of OLT, one subject was transplanted 9 days after his last dose of bevacizumab, fortunately without incident. This experience is difficult to directly compare with the contemporaneous phase II trial of single-agent bevacizumab in advanced HCC that reported an 11% incidence of serious bleeding, including one fatal event, because the advanced HCC study included a higher proportion of Child's class B subjects [26]. As the field moves forward with the evaluation of anti-angiogenic agents in early stage HCC, caution regarding the risk of bleeding and the potential for decreased wound healing in this special population is advised.

The PK parameters for bevacizumab observed in the small number of subjects (n = 15) were within the range expected for oncology subjects in general. The population PK model of bevacizumab was based on a heterogeneous population that included subjects with liver metastasis, yet liver function enzymes (e.g., alkaline phosphatase and serum glutamic oxaloacetic transaminase) were not covariates that significantly influenced bevacizumab PK. These clinical findings are substantiated by a preclinical study in rats in which hepatic dysfunction induced by bile duct ligation did not alter the exposure (AUC0 - 11) of bevacizumab following a single 20 mg/kg IV bolus injection [27]. Overall, based on these findings, well-compensated subjects with hepatocellular carcinoma are expected to have predictable bevacizumab disposition.

Serum VEGF levels increased after TACE in both study arms, as described in previous studies [10,12,17], but the administration of bevacizumab appeared to mitigate this effect. These results demonstrate that post-TACE VEGF levels can be modulated by bevacizumab, thereby reducing the ability of TACE to stimulate VEGF-mediated angiogenesis.

In summary, this study demonstrates that TACE combined with bevacizumab is safe and feasible in carefully-selected subjects with HCC. The size of the study limits any strong conclusions regarding efficacy, and ultimately a large randomized study would be required to determine if there is an improvement in outcome with the addition of bevaczicumab over TACE alone. Since this study was first designed and implemented, there have been many advances in the field, including confirmation of proof of principal for anti-angiogenic agents in advanced HCC [21], incorporation of novel imaging modalities to monitor angiogenesis in vivo [24], adaptation of standardized response criteria in HCC [25], and the development of doxorubicin-impregnated beads for TACE [28]. Larger ongoing studies with agents such as sorafenib and brivanib will help clarify the role of systemic anti-angiogenic agents as an adjunct to TACE. This pilot study, though limited by the state of the art at its inception, provides further support for this approach.

## Competing interests

This investigator-initiated study was funded by Genentech, Inc. C.D.B., Z.A.W., and R.S.F. have received honoraria from Genentech, Inc. Y.X. is an employee of Genentech, Inc. Y.X. has Roche stock.

## Authors' contributions

CDB participated in study design, recruited and followed study subjects, analyzed data, and drafted the manuscript. ASG participated in study design, performed and analyzed all angiograms, and revised the manuscript. ZAW recruited and followed study subjects, and revised the manuscript. DE performed statistical analysis. RA conceived of the study and participated in study design. YX performed the PK analysis and drafted the PK sections of the manuscript. RWB participated in study design, and recruited study subjects. DJS conceived of the study and participated in study design. RSF participated in study design, recruited and followed study subjects, analyzed data, and revised the manuscript. All authors read and approved the final manuscript.

## Pre-publication history

The pre-publication history for this paper can be accessed here:

http://www.biomedcentral.com/1471-2407/12/16/prepub
